# Grain-Size Distribution Effects on the Attenuation of Laser-Generated Ultrasound in α-Titanium Alloy

**DOI:** 10.3390/ma12010102

**Published:** 2018-12-29

**Authors:** Xue Bai, Yang Zhao, Jian Ma, Yunxi Liu, Qiwu Wang

**Affiliations:** 1 Laser Institute, Qilu University of Technology (Shandong Academy of Sciences), Jinan 250103, China; baixue0130@163.com (X.B.); majianem@163.com (J.M.); qwizard@live.com (Q.W.); 2 Department of Materials Research, Avic Manufacturing Technology Institute, Beijing 100024, China; liuyunxi9906@163.com

**Keywords:** grain-size distribution, laser ultrasonics, α-titanium alloy, ultrasonic attenuation

## Abstract

Average grain size is usually used to describe a polycrystalline medium; however, many investigations demonstrate the grain-size distribution has a measurable effect on most of mechanical properties. This paper addresses the experimental quantification for the effects of grain-size distribution on attenuation in α-titanium alloy by laser ultrasonics. Microstructures with different mean grain sizes of 26–49 μm are obtained via annealing at 800 °C for different holding times, having an approximately log-normal distribution of grain sizes. Experimental measurements were examined by using two different theoretical models: (i) the classical Rokhlin’s model considering a single mean grain size, and (ii) the improved Turner’s model incorporating a log-normal distribution of grain sizes in the attenuation evaluation. Quantitative agreement between the experiment and the latter model was found in the Rayleigh and the Rayleigh-to-stochastic transition regions. A larger attenuation level was exhibited than the classical theoretical prediction considering a single mean grain size, and the frequency dependence of attenuation reduced from a classical fourth power to an approximately second power due to a greater probability of large grains than the assumed Poisson statistics. The provided results would help support the use of laser ultrasound technology for the non-destructive evaluation of grain size distribution in polycrystalline materials.

## 1. Introduction

Average grain size (diameter) is usually used to describe a polycrystalline medium; however, many investigations demonstrate the grain-size distribution actually has a measurable effect on most mechanical properties, such as tensile strength [1], yield stress [2], flow stress [3,4], hardness [5], etc. For example, good ductility and high strength can be achieved for fine grains with a small distribution width after recrystallization for commercial pure titanium alloy [6], while exceptionally large grains can result in unacceptably low mechanical strength for a pure niobium sample [7]. Two specimens with the same mean grain size can have a significantly different distribution of grain sizes in most cases. However, the distribution of grain sizes can deviate from the design specifications during metallurgical processes such as heat treatment or machining. It is therefore highly desirable for the characterization of the distribution of grain sizes, especially in a non-destructive way.

When ultrasonic waves propagate through a polycrystal, inhomogeneities in elastic properties and in density lead to the variation of propagation velocities in each crystallite, consequently resulting in ultrasonic wave scattering. Thus, at least theoretically, grain size can be expected to be evaluated by measuring the scattering-induced ultrasonic attenuation. Theoretical foundations for the correlation of attenuation to grain size were laid out by two seminal works. The first one, developed by Stanke and Kino [8], was a unified framework based on a second-order perturbation theory [9]. A nonlinear equation for the wave propagation constant was obtained, applicable for a cubic equiaxial untextured polycrystalline medium and valid in all frequency regions. It reproduced the classical frequency dependence of attenuation in the Rayleigh and the stochastic regions [10,11]. The second model, by Weaver [12], derived a general solution in untextured cubic-symmetry polycrystals using the Dyson and Bethe–Salpeter equation. Under the use of the Born approximation, these two seminal models gave rise to the same solution of the attenuation coefficient [13]. They were further extended to some particular cases [14,15]. For instance, Rokhlin et al. [16] obtained explicit formulas for ultrasonic wave attenuation coefficients in polycrystals with hexagonal symmetry based on the Weaver’s model.

Nevertheless, the models mentioned above were developed based on the assumption of a Poisson distribution of grain sizes and supposed that the spatial correlation function had an inverse exponential form of a single mean grain size. A complete grain-size distribution is not considered, which would change the spatial correlation function model assumed in the derivation, and accordingly, has been shown to affect the acoustic response of materials, specifically ultrasonic attenuation [17,18,19,20,21]. An analytical foundation for the effects of the distribution of grain sizes on ultrasonic attenuation was laid out by Smith [21]. Then Nicoletti et al. [19,20] made an analytical investigation for the inverse problem, i.e., characterization of grain-size distributions from ultrasonic attenuation. More recently, within the context of the Weaver’s model, Turner et al. [22] provided analytical formulas for ultrasonic attenuation evaluation by incorporating the log-normal distribution of grain sizes, usually reported using microscopy studies of most real polycrystalline metals [2,17,23]. Their results were applicable for polycrystalline materials with arbitrary crystallite symmetry. Both the amplitude and the frequency dependence of attenuation were shown to vary with a distribution width of grain sizes, even for the same mean grain size, and it was suggested that grain size characterization through the inversion of attenuation would cause large errors if the distribution was neglected.

The ability to use ultrasonic attenuation for grain size characterization in polycrystalline materials has been demonstrated by experimental measurements [24,25,26,27,28]. Correlation of the experimentally measured attenuation to a single mean grain size are usually established. Nevertheless, some studies found the distribution of grain sizes could affect the accuracy of the experimental confirmation of the classical theories [7,27,28]. For example, Zhang et al. [27] carried out the measurements of the dependence of ultrasonic attenuation on mean grain size in copper samples in the Rayleigh frequency region. A linear dependence on the mean grain size of the attenuation level was observed, rather than a third power dependence as predicted by the classical models. They suspected the distribution of grain sizes, which deviates significantly from the assumption in the classical models, as one of possible reasons for the difference between theory and experiment. Otherwise, in the Rayleigh-to-stochastic transition region, Zeng et al. [7] found that experimental estimated attenuation in high purity niobium samples showed a much larger level than the one predicted by the Stanke–Kino unified model. The authors proposed that a great probability of large grains measured in the banded microstructure of fine and coarse grains perhaps could explain this difference.

The present work addresses the correlation of the grain size distribution to attenuation of laser-generated ultrasound. Experimental evaluation in a polycrystalline α-type titanium alloy with hexagonal close-packed crystal structure was examined by using two theoretical attenuation models: (i) the classical Rokhlin’s model considering a single mean grain size, and (ii) the improved Turner’s model incorporating a log-normal distribution of grain sizes. The novelty of this research is the experimental quantification for effects of grain-size distribution on the frequency and grain size dependence of ultrasonic attenuation, which validates the improved theory. Materials and experimental procedures are first introduced in the subsequent section, following by results on ultrasonic attenuation measurements and grain size characterization in Section 3. Discussions about comparison of experiment and theory on grain-size distribution effects on ultrasonic attenuation are finally made in the Section 4.

## 2. Materials and Methods

**Figure 1:**
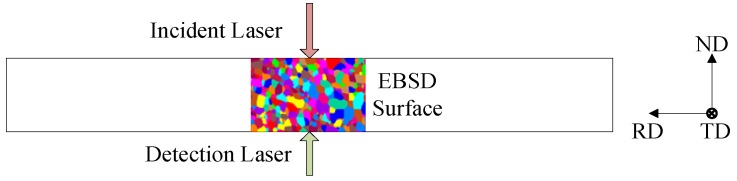
Schematic of specimen with the normal direction (ND) aligned with the ultrasonic wave propagation direction, the rolling direction (RD) corresponding to the length of the specimen, the transverse direction (TD) to the width, and the EBSD observations were made in the RD-ND plane in the region where the ultrasonic pulse propagates through. (Color online).

Titanium alloys are widely used in various areas because of the high specific strength, the wide operating temperature range, the strong corrosion resistance and the good biocompatibility, etc. [29,30]. In this preliminary investigation, commercially pure titanium TA2 (99.5 wt% Ti) plates were chosen, which were relatively clean of impurities, voids, and second phases. The contribution of scattering at grain boundaries was expected to dominate the ultrasonic attenuation and was accordingly easy to identify. The as-received material was in a cold-rolled condition, composed of elongated polygonal α grains with a mean size less than 15 μm. For the laser ultrasonic experiments, plate specimens having dimensions of 200 × 100 × 10 mm^3^ were cut, with the normal direction (ND) aligned with the smallest dimension and parallel to the wave propagation direction, and the rolling direction (RD) aligned with the largest dimension (Figure 1). One of them was used as a reference specimen, while five specimens were treated with a ZK-16QX-1400TP chamber furnace (Beijing Zhongke Beiyi Technology Co., Ltd., Beijing, China) at a constant temperature for different holding times, and then quenched in the air to room temperature. In order to avoid the influence of the phase transformation on the ultrasonic response, the holding temperature was set to 800 °C, below the β-transus (T = 882 °C) temperature, and holding times were set to 0.5 h, 1 h, 2 h, 4 h, and 8 h [6]. In the heating process, the mean grain size was expected to show an exponential growth as the holding time without phase transformation [6].

**Figure 2:**
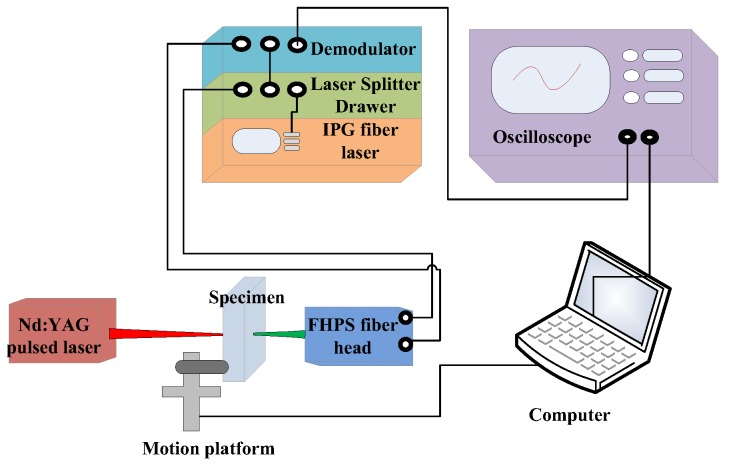
Schematic diagram of experimental system by laser ultrasonics. (Color online).

Concerning the ultrasonic inspections, a laser pulse was generated using a Q-switched Nd:YAG pulsed laser (Wuhan Lead Laser Co., Ltd., Wuhan, China) with a wavelength of 1064 nm. The maximum energy of the laser pulse was approximately 28 mJ and its duration was 10 ns. The pulsed laser spot was adjusted to a diameter of 1 mm. The incident laser pulse on the sample surface excited a broadband longitudinal ultrasound pulse in the ablative regime [23], which propagated back and forth in the ND. Then the transmitted pulse was detected by using the IOS AIR-1550-TWM laser ultrasonic receiver (Intelligent Optical Systems, Inc., Torrance, CA, USA) based on two-wave mixing in a photorefractive crystal at the opposite side of the sample surface. The detected signal was averaged 64 times on an oscilloscope and downloaded to a computer for analysis and processing. To avoid the detection of the other wave modes, the detection laser beams were colinearly aligned with the generation one, focusing at the epicenter of ultrasonic waves. It is worth noting that samples after annealing were polished for the ultrasonic inspection since a high surface reflection coefficient is required by the FHPS fiber head (Intelligent Optical Systems, Inc., Torrance, CA, USA) used in this experiment study. In the present configuration, the sample was fixed on a two-dimensional motion platform, by which the B-scan experiments along a specified path of a sample could be achieved (Figure 2). Through the time-domain signal processing based on a fast Fourier transform (FFT), a correlation of frequency dependence of ultrasonic attenuation with the variation in the grain-size distribution was established.

Destructive metallographic observations for samples were then carried out using electron back-scattering diffraction (EBSD) (TSL(EDAX), Mahwah, NJ, USA). The specimens were grinded and electrolytically polished with a finishing solution of 10% perchloric acid, 60% methyl alcohol, and 30% n-butyl alcohol in volume fraction at the current density of 100–150 A·dm^−2^. The observation step size used during EBSD scanning was 1.5 μm. In order to obtain the information on grains interacting with the ultrasonic pulse, the EBSD observations were made in the RD-ND plane in the region where the ultrasonic pulse propagated through (Figure 1). All the EBSD maps were ≥1.5 mm × 1.5 mm. At least 900 grains in total were measured at different locations for each microstructure. Microstructural information was obtained by using the orientation imaging microscopy (OIM) technique, such as the phase composition of the material, and the crystallographic orientation and area of each crystallite. The mean grain size $\bar{D}$ is calculated as (4$\bar{A}$/π)^0.5^ where $\bar{A}$ is the mean grain area.

## 3. Results

### 3.1. Metallographical Observations

**Figure 3:**
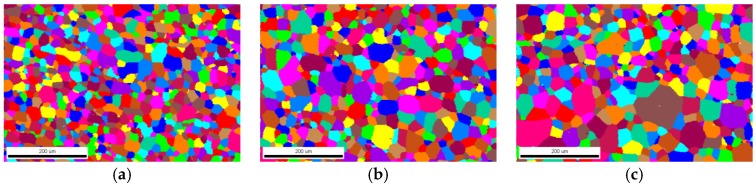

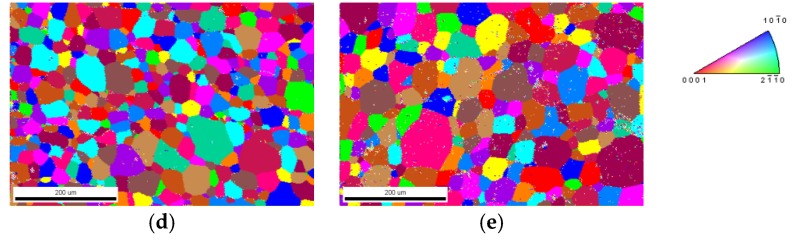
EBSD maps of commercially pure titanium showing the microstructure annealed at 800 °C holding for (**a**) 0.5 h, (**b**) 1 h, (**c**) 2 h, (**d**) 4 h, and (**e**) 8 h. (Color online).

Figure 3 shows the EBSD maps measured for the specimens heat-treated at 800 °C for different holding times. Data for each specimen in the EBSD investigation are presented in Table 1, including the fraction of the α-phase, the mean grain size $\bar{D}$, the distribution width of grain sizes and the number of sampled grains. Since the specimens are treated below the β-transus temperature, an insignificant number of β-phase crystallites were observed. Despite the fact that grains were deformed by rolling, metallographic observations show that the subsequent annealing treatments after holding for 0.5 h produced an approximately equiaxed crystallite with an aspect ratio of about 0.78.

As can be inferred from the evolution of the mean grain size and distribution width values, a heterogenous grain growth occurred during the isothermally annealing, rather than the general exponential growth relationship as predicted by the kinetic model of grain growth [6]. Specifically, within the first two hours, both the mean grain size and the distribution width of grain sizes showed a rapid growth. Hereafter, the mean grain size decreased at first, then increased slowly. Nevertheless, the dispersion degree of grain sizes decreased continuously with the holding time, indicating the grain size tended to be more homogeneous. After holding for 8 h, the final average grain size had increased by about twice.

To evaluate the grain-size distribution effects on the ultrasonic response, a careful characterization for the grain-size distribution in each specimen was made. Following a series of studies [2,3,4,17,18,19,20,21,22,23,31], a log-normal distribution function, which has been shown to closely represent the realistic polycrystalline microstructures, was then used to fit the grain size distribution data by using the Levenberg–Marquardt nonlinear least squares algorithm. According to the fitting model, the logarithm of the size *D* of each crystallite is normally distributed, i.e., ${\ln(D)\sim N(\mu ,\;\sigma }_{d}$, with μ and $\sigma_{d}$ donating the mean and the standard deviation. The probability density function for a log-normal grain size distribution reads as [22]:

(1) $$P(D)=\frac{1}{{D\;\sigma }_{d}\sqrt{2\pi }}\exp(-\frac{{\left(\ln D-\mu \right)}^{2}}{{2\sigma }_{d}^{2}}),$$

where the estimated mean grain size $\tilde{D}$ by the fitted model is defined as: $\tilde{D}$ = exp ($\sigma_{d}^{2}$/2 + *μ*).

**Figure 4:**
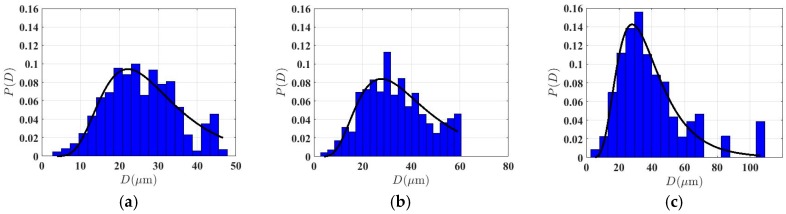

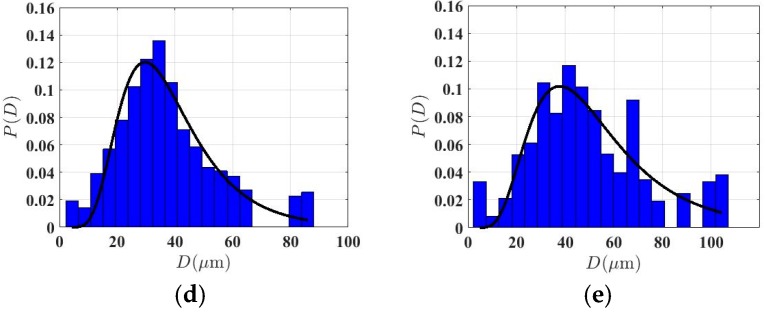
Grain size distributions determined from optical micrographs by EBSD investigations for specimens annealed at 800 °C holding for (**a**) 0.5 h, (**b**) 1 h, (**c**) 2 h, (**d**) 4 h, and (**e**) 8 h presented as blue histograms, with fitted curves for log-normal size distribution superposed in solid lines. (Color online).

Microstructure data of the grain-size distribution determined from optical micrographs are plotted as blue histograms in Figure 4, with the fitted curves for log-normal distribution presented as solid lines. Parameters for the fitted model with the root-mean-square error (RMSE) less than about 0.02 are listed for each microstructure in Table 1, such as the standard deviation $\sigma_{d}$ and the mean value μ. It is shown that the standard deviation value describing the dispersion degree of distribution of grain sizes ranged from 0.42 to 0.51 with an average of about 0.46. This is within the range of 0.33–0.76 for several polycrystalline materials as reported in the literature [2,17,22,23]. This would have a measurable effect on the frequency dependence of attenuation in the context of laser ultrasonic detection as predicted by the Turner’s model [22]. Furthermore, it was observed that the ratio of the estimated mean grain size $\tilde{D}$ to the one obtained by OIM analysis $\bar{D}$ was about 1.0–1.2. The difference between them may be attributed to the assumption of spherical grains used in the fitted model. Rigorous comparison shows that the fitted model underestimates the fraction of some exceptionally large grains produced by the anomalous grain growth, especially for the specimens with holding times greater than 2 h.

### 3.2. Ultrasonic Attenuation Measurements

**Figure 5:**
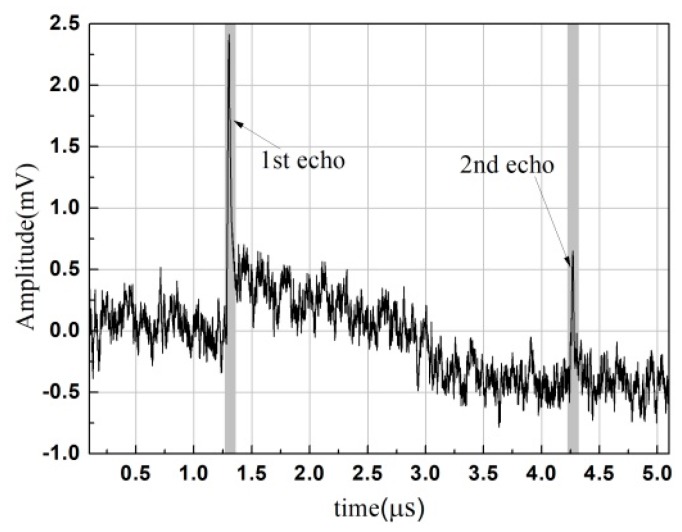
Laser-generated ultrasound waveform measured for α-titanium alloy annealed at 800 °C holding for 1 h.

Figure 5 shows a typical laser-generated ultrasound waveform measured for the specimen annealed at 800 °C holding for 1h. The first compressive echo signal observed at 1.3 µs corresponds to the initial pressure pulse having transmitted to the receive surface. After about 3 µs, the second echo arrived at the epicenter of the receive surface, which corresponds to the initial pressure pulse having transmitted to the receive surface for the second time. Concerning the oscillations before 1.3 µs and between the first and second compressive echoes, there were generally two main sources: one was the high-frequency noise in the system and the other was the noises backscattered by grain boundaries. Other wave modes of propagation were not measured since the detection point was at the epicenter.

**Figure 6:**
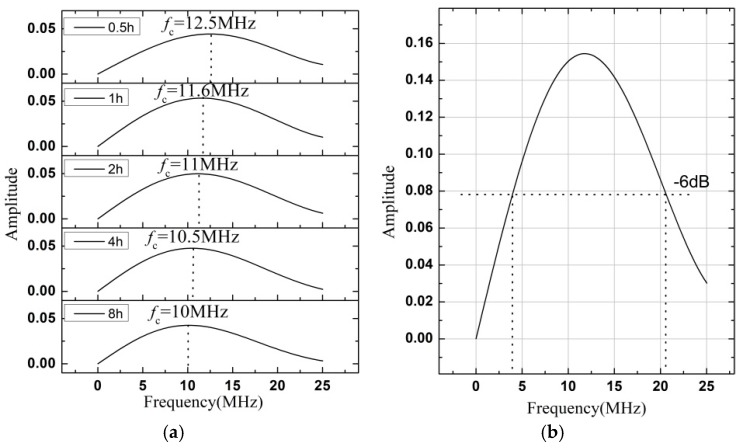
Frequency amplitude spectrum of the second compressive echoes measured after different holding times at 800 °C (**a**), and of the first compressive echo measured for the as-received sample (**b**).

The maximum amplitude of each compressive echo was centered in a window with a width of 0.1 µs. By setting the time gate, the time-domain signal of each compressive echo (shown as the gray parts in Figure 5) were acquired. After filtering the direct current weight and the high-frequency noises, their amplitude spectra were calculated by using the FFT algorithm for both the first and second compressive echoes. Results for the second ones are plotted, as an example, in Figure 6a. The central frequency *f*_c_ is presented as vertical dot lines, and varied between 10 MHz and 12.5 MHz with the holding times. It seemed to decrease with the grain growth during isothermal annealing. Further study needs to be done to understand this phenomenon. In the present case, according to the amplitude spectrum of the first compressive echo for the as-received reference sample, the valid frequency bandwidth was given as being approximately between 4 and 20 MHz, i.e., for wavelengths ranging from about 300 to 1500 μm (Figure 6b).

For a longitudinal wave propagating in the polycrystalline medium, three sources of the measured ultrasonic attenuation mainly exist [28,32]. The most important source arises from attenuation by scattering, $\alpha_{s}$, and is attributed to interactions between waves and grain boundaries due to inhomogeneities in elastic properties or in density between adjacent grains, which is of interest in the present case. The second contribution is associated with the diffraction of ultrasound pulse, $\alpha_{d}$, and seems to depend on the geometry of the specimen and the propagation distance. The third one is involved with attenuation by internal friction and is frequency-independent. It is generally negligible with respect to the scattering phenomenon [28,33]. Generally, for a longitudinal wave propagating along the *z* direction from ${z=z}_{1}$ to ${z=z}_{2}$, the total attenuation per unit length, expressed in dB/mm, is evaluated by comparing the amplitude spectrum *A* measured in each waveform:

(2) $$\alpha_{\mathrm{total}}{(\;f\;)\;=\;\alpha }_{s}{+\alpha }_{d}{+\alpha }_{f\;}=\frac{20}{z_{2}{-z}_{1}}\log_{10}\frac{{A(z}_{2},\;f\;)}{{A(z}_{1},\;f\;)}\;,$$

For measuring the grain boundary contribution to the attenuation with high accuracy, it is crucial to exclude the impacts of the other two sources. Herein, the attenuation by diffraction, $\alpha_{d}$, can be estimated by using the as-received sample having the same geometry and the same propagation distance and having insignificant scattering [28,32,34]. Under this assumption, an attenuation coefficient, $\alpha (\;f\;)=\alpha_{\mathrm{total}}(\;f\;)\;-\alpha_{d}$, is then calculated by subtracting the total attenuation of the as-received specimen from the one of the studied medium. It can be generally composed of two parts: an insignificant frequency-independent term, *a*, mainly accounting for the internal friction, and a frequency-dependent term associated with scattering by grains which is assumed proportional to $\bar{D}$^*n*−1^*f^n^* [8,12]. Thus, α can be expressed as follows [28,32,34]:

(3) $$\alpha (f)=a+b{\bar{D}}^{n-1}f^{n},$$

where, *b* is a parameter determined by the material’s properties. It is known from classical theories [8,12], 0 ≤ n ≤ 4 depending on the ratio of ultrasonic wavelength to the grain size: *x*_0_ = 2π$\bar{D}$/*λ* [8]. Equation (3) is used to fit on the frequency and grain size dependency of attenuation in the following.

**Figure 7:**
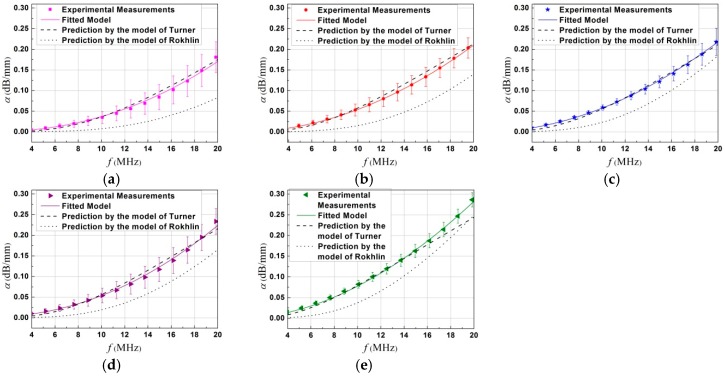
Comparison in attenuation coefficient versus frequency between experimental estimations and two different theoretical models: (i) the Rokhlin’s model [16] considering a single mean grain size, and (ii) the improved Turner’s model [22] incorporating a log-normal distribution of grain sizes, for a longitudinal wave in α-titanium alloy annealed at 800 °C holding for (**a**) 0.5 h, (**b**) 1 h, (**c**) 2 h, (**d**) 4 h, and (**e**) 8 h. The solid curves correspond to fitted results using an approximate power function in Equation (3). (Color online).

Experimental measurements of the longitudinal wave attenuation coefficient are shown with scatter markers in Figure 7. For each specimen, this was measured using the average response over several waveforms from different measure points. The standard deviation resulting from different measurements in the same sample is presented by the vertical error bars. It was observed that, for a given mean grain size, not only the attenuation but also the deviation level from the average between different measurements increased with frequency.

On the basis of the data on metallographic-determined grain sizes, an approximate power function as shown in Equation (3) is used to fit on experimental measurements of attenuation versus frequency by means of the nonlinear least squares. Results of fitting are shown in Figure 7 with solid curves and parameters for the evaluation function with 95% confidence bounds are displayed in Table 2. These fitted curves exhibit a goodness of fit with the coefficients of multiple determination all above 0.99 and the RMSE value below 0.005. The attenuation was shown to have a power law dependence on frequency with a mean value of 2.0 (*n* = 1.86–2.20), which is close to the classical theoretical prediction in the stochastic scattering region. Nevertheless, when the range of values 2π$\bar{D}$ was compared to the ultrasonic wavelengths employed, the Rayleigh scattering was indeed expected for most of samples, and would exhibit a fourth power law dependence on frequency as predicted by the classical theories [8,12,16]. Effects of grain size distribution on the reduce of frequency dependence of attenuation will be further evaluated in the following section.

### 3.3. Grain Size Characterization

**Figure 8:**
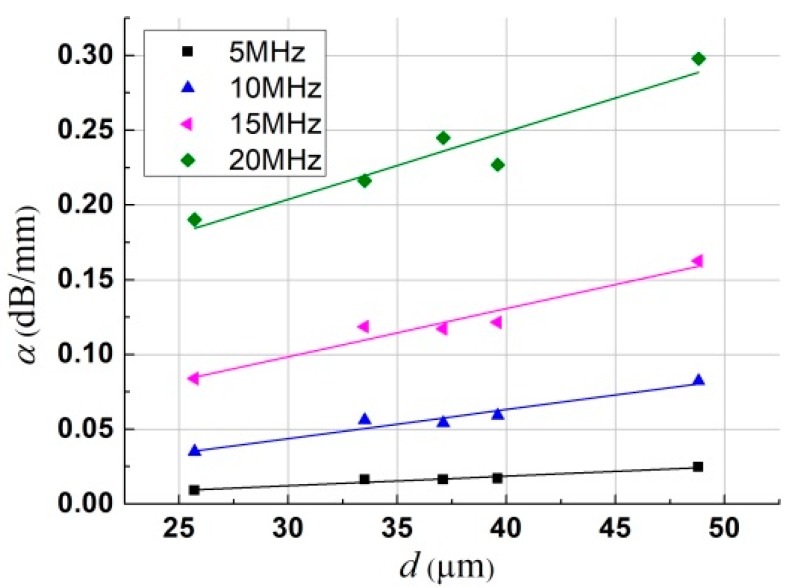
Linear fitting for grain size dependence of attenuation at four given frequencies. (Color online).

The grain size dependency of attenuation was further studied for the application to nondestructive grain size measurement. According to the power law of attenuation on frequency obtained above, the measured attenuation was used to establish the linear relationship of grain size at the given frequencies of 5, 10, 15, and 20 MHz, and the attenuation data are replotted as a function of grain size in Figure 8. The coefficients of multiple determination of linear fitting were all above 0.932, showing a good fit. The linear dependence of attenuation on grain size was in good agreement with results in the recent literature [7,35], which can be further used to approximately predict the mean grain size by inversion of laser ultrasonics.

## 4. Discussions

In order to investigate the effects of the grain size distribution on ultrasonic attenuation, we made a comparison of the experimental estimations with (i) the Rokhlin’s model considering a single mean grain size [16], and (ii) the improved Turner’s model incorporating a log-normal distribution of grain sizes in the attenuation evaluation [22]. Following classical theories [8,16,22], the attenuation coefficient of longitudinal wave, $\alpha_{L}$, can be expressed as the sum of two terms: one induced by the scattering into the same type of wave $\alpha_{\mathrm{LL}}$, and the other one generated by the mode conversion into the transverse wave mode $\alpha_{\mathrm{LT}}$,

(4) $$\alpha_{L}=\alpha_{\mathrm{LL}}+\alpha_{\mathrm{LT}}$$

where the subscripts “*L*” and “*T*” specify the longitudinal and the transverse waves respectively. Expressions for these components are different between the two cases of a single mean grain size and a log-normal distribution of grain sizes. Considering hexagonal polycrystalline titanium alloys with a single mean grain size, theoretical results for attenuation coefficients were evaluated by the explicit formulas in the work of Rokhlin et al. [16] as follows:

(5) $$\alpha_{\mathrm{IS}}=\frac{{8\pi }^{4}f^{4}a^{3}}{V_{I}^{3}\rho^{2}}\frac{\Omega_{\mathrm{IS}}}{V_{s}^{5}}$$

with

(6) $$\Omega_{\mathrm{IS}}=\frac{2A_{\mathrm{IS}}+2B_{\mathrm{IS}}R_{\mathrm{IS}}^{2}+2C_{\mathrm{IS}}R_{\mathrm{IS}}^{4}}{b_{\mathrm{IS}}^{2}(R_{\mathrm{IS}}^{2}-1)}-\frac{2B_{\mathrm{IS}}R_{\mathrm{IS}}+4C_{\mathrm{IS}}R_{\mathrm{IS}}^{3}}{b_{\mathrm{IS}}^{2}}\ln(\frac{R_{\mathrm{IS}}+1}{R_{\mathrm{IS}}-1})+\frac{6C_{\mathrm{IS}}R_{\mathrm{IS}}^{2}}{b_{\mathrm{IS}}^{2}}+\frac{6B_{\mathrm{IS}}+2C_{\mathrm{IS}}}{{3b}_{\mathrm{IS}}^{2}},\;a_{\mathrm{IS}}=1+{{(k}_{I}^{2}a)}^{2}+{{(k}_{s}^{2}a)}^{2},\;\;b_{\mathrm{IS}}=2k_{I}k_{s}a^{2},\;{\;R}_{\mathrm{IS}}=a_{\mathrm{IS}}/b_{\mathrm{IS}},$$

where, the subscripts “*I*” and “*S*” denote the incident and scattered wave modes respectively, and *IS* = *LL* or *LT* for the case of the longitudinal wave. $V_{\xi }$ and $k_{\xi }$ denote the wave phase velocity and the propagation constant for the longitudinal wave (ξ=L) and the transverse wave (ξ=T), and ρ denotes the density. Here, 2*a* = $\bar{D}$ which corresponds to the mean cord length of crystallites [8,16]. The coefficients $A_{\mathrm{IS}}$, $B_{\mathrm{IS}}$ and $C_{\mathrm{IS}}$ only related with the elastic constants have been defined in [16]. The grain size was assumed to be equiaxed and have a distribution of Poisson statistics following the Weaver’s scattering model; therefore, the spatial correlation function W(r) could be approximated using an inverse exponential function: *W*(*r*) = exp(−2*r*/$\bar{D}$), describing the probability that two points at distance *r* fall in the same grain. Whereas, for a continuous log-normal distribution of grain sizes, the spatial correlation function may be written as follows [22]:

(7) $$W(r)=\int_{0}^{\infty }P(D)\exp(-r/D)\mathrm{dD},$$

where the probability density function of grain size distribution P(D) is defined by Equation (1). On the basis of parameters obtained from the best-fit log-normal distribution function shown in Table 1, the Equations (8) and (9) were then used to solve numerically the attenuation coefficients in all the considered samples following the work of Turner et al. [22]:

(8) $$\alpha_{\mathrm{IS}}=\frac{k_{s}^{4}\pi }{{4V}_{I}^{3}V_{s}\rho^{2}}\int_{0}^{\pi }{\tilde{\eta }}_{IS}{(\theta }_{\mathrm{ps}}{)M}_{\mathrm{IS}}{(\theta }_{\mathrm{ps}}{)\sin\theta }_{\mathrm{ps}}{d\theta }_{\mathrm{ps}}$$

with

(9) $${\tilde{\eta }}_{IS}{(\theta }_{\mathrm{ps}})=\int_{0}^{\infty }P(D)\frac{D^{3}}{\pi^{2}{\left[1+D^{2}\left(k_{I}^{2}{+k}_{s}^{2}+{2k}_{I}k_{s}{\cos\theta }_{\mathrm{ps}}\right)\right]}^{2}}\mathrm{dD},$$

where ${\tilde{\eta }}_{IS}$ denotes the spatial Fourier transform of the spatial correlation function, $\theta_{\mathrm{ps}}$ is the scattering angle. $M_{\mathrm{IS}}$ is related to the autocorrelation function of elastic constants for the corresponding incident and scattered wave modes and has been defined in [22]. The elastic constants used in both of the calculations were: *C*_11_ = 162, *C*_12_ = 92, *C*_13_ = 69, *C*_33_ = 180, *C*_44_ = 46.7 in GPa and the density was ρ= 4.5 g/cm^3^, and the two independent elastic constants of the effective isotropic medium were taken to be the Voigt average of the elastic constants of individual grains: $C_{11}^{0}=(8C_{11}+4C_{13}+3C_{33}+8C_{44})/15$, and $C_{44}^{0}=(7C_{11}-{5C}_{12}-{4C}_{13}+{2C}_{33}{+12C}_{44})/30$ [36].

**Figure 9:**
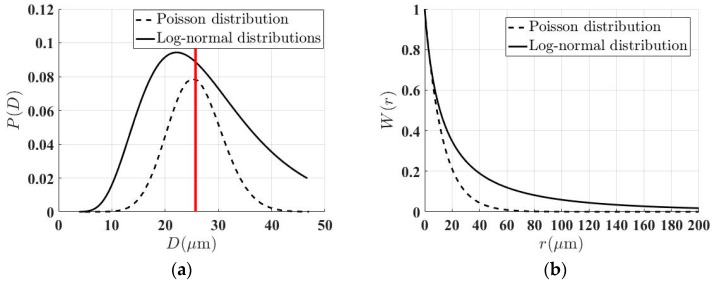
Comparison in (**a**) the probability density function of grain-size P(D), and (**b**) the spatial correlation function W(r) between the assumed Poisson statistics in the classical model by Rokhlin and the log-normal distribution model fitted for the specimen holding for 0.5 h with a mean grain size of $\bar{D}$ = 26 μm. (Color online).

It is seen from Figure 7 that our experimental data was quantitatively larger than the theoretical prediction by the Rokhlin’s model considering a single mean grain size, while it is in reasonable agreement with the one by the Turner’s model incorporating a closely log-normal distribution of grain sizes at all the frequencies. In order to provide a physical interpretation on this phenomenon, the specimen holding for 0.5 h with a mean grain size of $\bar{D}$ = 26 μm is taken as an example, and its fitted model of log-normal size distribution is compared to the Poisson statistics assumed in the Rokhlin’s model in both the probability density function of grain sizes P(D) (Figure 9a) and the spatial correlation function W(r) (Figure 9b). It is known that the larger grains seem to dominate the attenuation at a given frequency [7,28]. Indeed, a greater probability of large grains with a log-normal distribution was found compared to the idealized Poisson distribution assumed by Rokhlin, which led to a larger value of spatial correlation function at a given distance *r*. This maybe explains why a greater attenuation level was observed than the predicted attenuation with a single mean grain size.

Rigorous comparison shows that both of theoretical prediction underestimated the experimental data for the sample with the largest grain size of 49 μm in the high frequency region of 15–20 MHz. In fact, careful observations of grain size distribution in Figure 4 show that the fitted log-normal distribution function did not completely cover the real distribution of grain sizes. For instance, for the sample with the largest grain size of 49 μm, approximately 4% of large grains of about 70 μm and 5% grains of 100 μm were not considered in the fitted grain size distribution model. This value was relatively larger than the other four samples, which maybe provides information on possible reasons for this disagreement at high frequencies.

**Figure 10:**
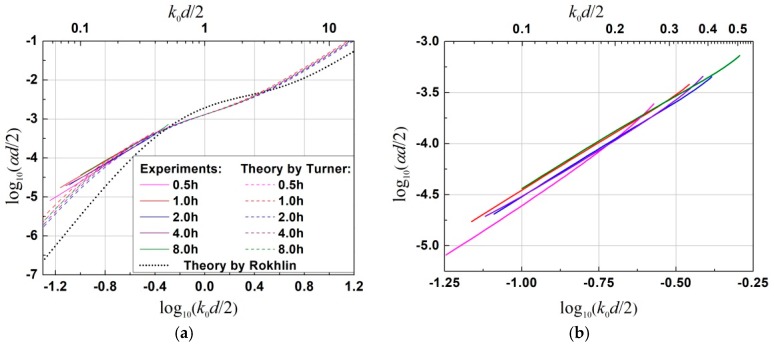
(**a**) Comparison in attenuation coefficient versus frequency by using the logarithmic scale, log_10_(*α*$\bar{D}$/2) vs. log_10_(*k*_0_$\bar{D}$/2), between experimental estimations and two different theoretical models: (i) the Rokhlin’s model [16] considering a single mean grain size, and (ii) the improved Turner’s model [22] incorporating a log-normal distribution of grain sizes, for a longitudinal wave in α-titanium alloy annealed at 800 °C after different holding times. (**b**) Zoom-in on experimental data for all the specimens that are roughly superposed. (Color online).

In order to further compare the frequency dependence of attenuation between the classical prediction and the experimental study, the logarithmic scale of normalized attenuation per length, *α*$\bar{D}$/2, versus normalized frequency, *k*_0_$\bar{D}$/2, is plotted in Figure 10a, where $k_{0}$ is the wave number of the effective medium. As predicted by the classical theoretical model [8,12], these curves were expected to be independent of grain sizes. Nevertheless, due to a slightly different distribution width of grain sizes, an insignificant deviation between samples was observed for data with a fitted log-normal grain size distribution.

It was seen that all the experimental attenuation data fell within the Rayleigh scattering and the Rayleigh-to-stochastic scattering transition regions. We can tell from the slope of the curves that the frequency dependence of attenuation measured by our experiments is in good agreement with the one predicted by theoretical model in consideration of a log-normal distribution of grain sizes. It exhibits close to a quadratic frequency dependence, rather than a close to quartic one as predicted by the classical theoretical model considering only a single mean grain size. Careful comparison between different specimens is made in Figure 10b by zooming in on the experimental data. As can be seen, the frequency dependence of attenuation seemed to be influenced by the size distribution width. Specifically, the specimen holding for 1 h and 8 h with a wider size distribution ($\sigma_{d}=0.50$ and 0.48 obtained from Table 1) seemed to have a higher normalized attenuation level and a lower slope. By contrast, the specimen holding for 0.5 h with the smallest size distribution width (*σ_d_* = 0.42) showed the lowest attenuation level but a largest slope for most of frequencies. However, a quantitative comparison seemed to be impossible since not only the distribution width but also the mean grain size was different. Furthermore, experimentally measured attenuation coefficients for all the samples deviated slightly from the theoretical prediction in consideration of a log-normal distribution of grain sizes in the low frequency region of log_10_(*k*_0_$\bar{D}$/2) < −1.0. Further investigations are to be made to understand this phenomenon.

## 5. Conclusions

Grain size distribution impacts on the attenuation of laser-generated ultrasound was evaluated in α-titanium alloys composed of grain sizes following a closely log-normal distribution with different standard deviation values of 0.42–0.50 and mean grain sizes of 26–49 μm. Experimental measurements were then examined by using two different theoretical models: (i) the classical Rokhlin’s model considering a single mean grain size, and (ii) the improved Turner’s model incorporating a log-normal distribution of grain sizes. Both the amplitude and the frequency dependence of attenuation were in good agreement with the ones predicted by the latter model. Polycrystals with a closely log-normal distribution of grain sizes showed a larger ultrasonic attenuation level than the theoretical prediction considering a single mean grain size, and the frequency dependence of attenuation was reduced from the classical fourth power to a close to second power. The attenuation accordingly shows an approximately linear relationship with grain size. Indeed, these differences are mainly attributed to a greater probability of large grains compared to the assumed size distribution in the classical model. Experimental estimations indicated that the larger the distribution width of grain sizes is, the smaller the frequency dependence seems to be. It would provide experimental basis for the non-destructive evaluation of grain size distribution in polycrystalline materials using laser ultrasonic technology.

## Figures and Tables

**Table 1:** Microstructure data obtained from the EBSD investigation and the parameters for the fitted model of log-normal size distribution.

Annealed Temperature/Holding Time	Data Obtained by OIM Analysis				Best-fit Log-normal Distributions			
	Fraction of α-phase	$\bar{D}$ (μm)	Distribution Width	Grain Sampled	$\sigma_{d}$	μ	$\tilde{D}$ (μm)	RMSE (10^−2^)
800 °C/0.5 h	1.000	26	9.3	1668	0.42	−10.24	29	1.44
800 °C/1.0 h	0.999	33	12.5	1050	0.50	−10.54	40	1.34
800 °C/2.0 h	0.999	39	14	1154	0.46	−10.27	39	1.51
800 °C/4.0 h	0.992	37	12.3	1150	0.43	−10.25	39	1.40
800 °C/8.0 h	0.987	49	10.7	916	0.48	−9.95	53	2.04

**Table 2:** Fit parameters of the approximate evaluation function $\alpha (f)=a+b{\bar{D}}^{n-1}f^{n},$ with b* = *b*${\bar{D}}^{n-1}$ obtained from the attenuation vs. frequency data in Figure 7.

Samples	$\bar{D}$ (μm)	*a* (10^−3^ dB/mm)	b* (10^−15^ dB·S*^n^*/mm)	*n*	R-square	RMSE (10^−2^ dB/mm)
0.5 h	26	1.60	0.015	2.20	0.9932	0.44
1.0 h	33	0.59	2.38	1.90	0.9996	0.14
2.0 h	39	1.08	1.86	1.93	0.9992	0.18
4.0 h	37	1.53	0.13	2.09	0.9964	0.41
8.0 h	49	1.59	7.07	1.86	0.9991	0.27
